# Post-COVID Syndrome: An Insight on Its Pathogenesis

**DOI:** 10.3390/vaccines9050497

**Published:** 2021-05-12

**Authors:** Helena C. Maltezou, Androula Pavli, Athanasios Tsakris

**Affiliations:** 1 Directorate of Research, Studies and Documentation, National Public Health Organization, 11523 Athens, Greece; 2 Department of Travel Medicine, National Public Health Organization, 11523 Athens, Greece; androulapavli2017@gmail.com; 3 Department of Microbiology, Medical School, National and Kapodistrian University of Athens, 15772 Athens, Greece; atsakris@gmail.com

**Keywords:** COVID-19, SARS-CoV-2, inflammation, post-infectious, complications, long term, pathogenesis

## Abstract

Post-COVID syndrome is increasingly recognized as a new clinical entity in the context of SARS-CoV-2 infection. Symptoms persisting for more than three weeks after the diagnosis of COVID-19 characterize the post-COVID syndrome. Its incidence ranges from 10% to 35%, however, rates as high as 85% have been reported among patients with a history of hospitalization. Currently, there is no consensus on the classification of post-COVID syndrome. We reviewed the published information on post-COVID syndrome, putting emphasis on its pathogenesis. The pathogenesis of post-COVID syndrome is multi-factorial and more than one mechanism may be implicated in several clinical manifestations. Prolonged inflammation has a key role in its pathogenesis and may account for some neurological complications, cognitive dysfunction, and several other symptoms. A multisystem inflammatory syndrome in adults (MIS-A) of all ages has been also described recently, similarly to multisystem inflammatory syndrome in children (MIS-C). The post-infectious inflammatory pathogenetic mechanism of MIS-A is supported by the fact that its diagnosis is established through serology in up to one third of cases. Other pathogenetic mechanisms that are implicated in post-COVID syndrome include immune-mediated vascular dysfunction, thromboembolism, and nervous system dysfunction. Although the current data are indicating that the overwhelming majority of patients with post-COVID syndrome have a good prognosis, registries to actively follow them are needed in order to define the full clinical spectrum and its long-term outcome. A consensus-based classification of post-COVID syndrome is essential to guide clinical, diagnostic, and therapeutic management. Further research is also imperative to elucidate the pathogenesis of post-COVID syndrome.

## 1. Introduction

More than a year after the declaration of the coronavirus disease 2019 (COVID-19) pandemic, the world continues to face its devastating impact, not only on morbidity, mortality, and healthcare services, but also its tremendous societal and economic consequences, globally [1]. Although the overwhelming body of knowledge on COVID-19 focuses almost exclusively on acute illness [2,3,4], it has become evident that long-term consequences occur [5,6].

Post-COVID syndrome was described for the first time in spring 2020 in the context of a survey of prolonged COVID-19 symptoms, run by the Patient-Led Research Collaborative, citizen’s scientist group [7]. Soon after the first COVID-19 cases evolved, they observed that COVID-19 patients had symptoms persisting for several weeks after acute infection [7]. The most common post-COVID symptoms include fatigue, dyspnea, olfactory and gustatory dysfunction, chest pain, myalgia, and sleep and mental disorders [6,7,8,9,10]. Symptoms may last for several months and disrupt work activities and the quality of life of affected individuals [6]. In recent months, our knowledge on post-COVID syndrome has expanded, mainly due to the recognition of new clinical manifestations, including rare neurological and thromboembolic complications, while the long-term consequences of the disease remain largely unknown [7,8,9,11,12,13]. It is estimated that 10% to 35% of patients not requiring hospitalization develop post-COVID symptoms, regardless of co-morbidities [6,14,15], while incidence rates up to 80% have been reported among hospitalized patients and among patients with severe illnesses [16,17,18].

Although there are published reviews on post-COVID syndrome [19,20,21], to the best of our knowledge, there are no reviews focusing on its pathogenesis. Understanding the underlying pathogenetic mechanisms of post-COVID syndrome is essential to guide evidence-based therapeutic management, but also to trigger future research. The purpose of this article is to provide an overview of the current state of knowledge regarding post-COVID syndrome, putting emphasis on its pathogenesis.

## 2. Materials and Methods

The PubMed was searched for articles published as of 30th Μarch 2021, using combinations of the following words: COVID-19, SARS-CoV-2, long-term, complications, post-COVID syndrome, chronic fatigue syndrome, pathogenesis, immune response, markers, chest pain, dyspnea, and psychological problems. We read the abstracts of a total of 164 articles and fully reviewed 98 articles. We also included information from official public health and scientific websites. Finally, 79 articles were included in the review, based on their relevance to the studied topic. Articles on children and adolescents < 18 years old were not considered.

## 3. Definition of Post-COVID Syndrome

Currently, there is no universally accepted definition of post-COVID syndrome. Post-COVID syndrome was defined for the first time by Greenhalgh et al. as COVID-19 associated illness extending for more than three weeks after the onset of symptoms, and chronic COVID-19 as persistent symptoms extending beyond 12 weeks after the onset of symptoms [7,14]. Recently, Amenta et al. proposed to the definitions outlined by Greenhalgh et al. that for patients who remain hospitalized at three weeks after symptom onset, the post-acute period starts when the patient is discharged from inpatient acute care [22].

## 4. Classification of Post-COVID Syndrome

According to the proposed criteria of the University of Cincinnati Medical Center for COVID-19 sequelae, there are five categories of long COVID-19 syndrome, based on initial symptoms, time of onset, and duration of symptoms and period of quiescence (Table 1): Type 1 includes patients with a varying duration of recovery that directly relates to the severity of the acute infection, organ complications, and underlying medical conditions; Type 2 is characterized by symptoms persisting six weeks from the onset of illness; Type 3 shows a period of quiescence or nearly full recovery, followed by a recurrence of symptoms persisting for at least three months (Type 3A) or at least six months (Type 3B); Type 4 refers to patients who are initially asymptomatic at the time of a positive SARS-CoV-2 test but become symptomatic one to three months (Type 4A), or at least three months later (Type 4B); and Type 5 includes patients who are asymptomatic or have few symptoms at the time of diagnosis and die within the next 12 months [18]. Amenta et al. from Baylor College of Medicine, Houston, classified post-acute COVID-19 manifestations in three categories, of which the first two should not be regarded as mutually exclusive: (1) residual symptoms that persist after recovery from acute infection; (2) organ dysfunction that persists after initial recovery; and (3) new symptoms or syndromes that develop after initial asymptomatic or mild infection [22]. Lastly, Fernandez-de-Las Penas et al. considered also undiagnosed cases and proposed a time-based classification as follows: potentially infection-related symptoms (up to 4–5 weeks), acute post-COVID symptoms (from week 5 to week 12), long post-COVID symptoms (from week 12 to week 24), and persistent post-COVID symptoms (lasting more than 24 weeks). Intrinsic and extrinsic predisposing factors are also considered [23]. A consensus-based standardization of definition and classification of post-COVID syndrome is needed to provide a common denominator for diagnostic and therapeutic approaches, but also for research purposes.

## 5. Clinical Manifestations

Post-COVID syndrome may encompass a plethora of debilitating symptoms and conditions [5,16,24,25,26,27,28,29,30,31,32,33]. Incidence of specific symptoms may range according to the severity of the acute infection and observation period. Fatigue is the most common post-COVID symptom, with an incidence ranging from 17.5% to 72% among hospitalized patients, extending on several occasions beyond seven months after the onset of illness and causing significant disability [6,17,24,25,26,29,31,34,35,36]. Dyspnea and a decreased exercise tolerance have been reported in 10–40% of hospitalized COVID-19 patients for 2–4 months after discharge [36,37], while new or deteriorating dyspnea was experienced by 65.6% of patients admitted to the intensive care unit (ICU) [34]. Chest pain has been reported in up to 22% of COVID-19 patients two months after discharge from hospital [16,24,26,35]. Gastrointestinal symptoms may persist in up to 30% of patients two months after hospital discharge [17,24,31]. Olfactory and gustatory dysfunction may extend beyond one month after its onset [27,28,38] and may affect up to 11% and 9% of patients six months after discharge from hospital, respectively, and up to 9% and 3.7% of patients eight months after mild COVID-19, respectively [6,26]. Cardiac arrhythmias and persistently elevated blood pressure are also reported after acute COVID-19 [39,40].

Sleeping and mental disorders, such as anxiety and depression, may affect approximately 26% and up to 40% of patients, respectively, even six months after COVID-19 [24,41]. Manifestations may include obsession and compulsion, reduced social activity, poor concentration, aggression, irritability, substance use, and cognitive deficit [37]. Post-traumatic stress disorder; a psychiatric condition induced by life-stressing factors, could occur after recovery from a life-threatening illness, including COVID-19; as shown by recent studies, the prevalence rate may range from 5.8% to 43% [25,41,42,43]. Neurological post-COVID complications have also been reported, including late-onset Guillain-Barré syndrome, opsoclonus-myoclonus syndrome, acute transverse myelitis. and cerebrovascular disorders, such as ischemic stroke, cerebral vasculitis and hemorrhage [44,45,46,47,48,49], and irreversible hearing loss [11], however, these severe conditions are rare [47]. Several psychiatric and neurologic conditions that are observed in post-COVID syndrome may also be linked with Alzheimer’s disease [50]. These include post-traumatic stress disorder, Guillain-Barré syndrome, depression, and cognitive impairment [50]. The worsening of motor function (51.9%) and an increased levodopa daily dose requirement (48.2%), followed by fatigue (40.7%) and cognitive and sleep disturbances (22.2% each), were present in 23 (85.2%) of 27 patients with Parkinson’s disease and COVID-19, who developed post-COVID syndrome [51].

Beyond clinical manifestations, a recent prospective study from the United Kingdom showed evidence of the physiological basis of post-COVID syndrome with measurable organ impairment [52]. In this study, 201 low-risk patients (mean age: 44 years, 71% female), with ongoing symptoms (mainly fatigue, shortness of breath, muscle ache, and headache) at least 4 weeks following recovery from acute infection were investigated through MRI, four months after initial symptoms [52]. Reference ranges derived from age-matched controls were used to evaluate the MRI findings [52]. The MRI investigation revealed impairment in at least one organ in 70% of cases, particularly in the heart in 26% (myocarditis: 19%, systolic dysfunction: 9%), lungs in 11% (reduced vital capacity), kidney in 4% (inflammation), liver in 28% (12% inflammation, 21% ectopic fat, 10% hepatomegaly), pancreas in 40% (15% inflammation, 38% ectopic fat), and spleen in 4% (splenomagely) [52]. Longer-term follow-up of organ function in the context of post-COVID syndrome is needed.

Beyond persisting symptoms, current evidence suggests that the overwhelming majority of patients with post-COVID syndrome show improvement over time and have a good prognosis with no further sequelae [29]. However, the full range, duration, and long-term outcome of post-COVID syndrome are largely unknown.

## 6. Pathogenesis of Post-COVID Syndrome

COVID-19 is a multi-system infection [53]. The cell surface angiotensin-converting enzyme 2 (ACE2) receptor, which is abundant in cells of most organs, is the main target for SARS-CoV-2 binding and infection [54,55]. A monocyte-macrophage, CD4 and CD8 cellular response, and a controlled inflammatory response occur, which results in an uncomplicated recovery of most patients [53]. A SARS-CoV-2 immune dysregulation, associated with elevated levels of cytokines interleukin-1β (IL-1β), IL-6, IL-2, and IL-10 (“cytokine storm”) and profound inflammation, is found in patients with severe life-threatening illnesses [53].

The pathogenesis of post-COVID syndrome remains largely unknown. Evidence suggests that prolonged inflammation has a key role in the pathogenesis of most post-COVID manifestations. Ortelli et al. recently studied 12 patients (median age: 67 years), who had recovered from COVID-19 with neurological complications and who complained of fatigue (median time from onset of COVID-19: 12 weeks; range: 9–13 weeks) [55]. All 12 patients had a hyper-inflammatory acute phase (markedly elevated C-reactive protein CRP and IL-6) [56]. The authors used neuropsychological and neurophysiological investigations, in comparison to 12 matched by age and gender healthy subjects, and found evidence for central abnormal neuromuscular fatigue, impaired cognitive control, reduced global cognition, apathy, and executive dysfunction in the post-COVID period, affecting their daily life [56]. Alteration of neuronal function in the context of the profound increase of circulating cytokines, and particularly IL-6, which can penetrate the blood-brain barrier, may occur and contribute to central nervous system (CNS) complications (e.g., altered mental status and neurocognitive disorders among others) [54]. In addition, COVID-19-associated inflammation might lead to Gamma–aminobutyric acid (GABA)-ergic impairment, possibly representing the basis of neuromotor and cognitive fatigue, and explaining apathy and executive deficits [57]. Indeed, animal models have shown that an IL-6 hyper-inflammatory-induced state may decrease the density of GABA receptors [57].

In another study conducted to elucidate the underlying mechanism of neurological complaints and cognitive dysfunction in post-COVID syndrome, plasma from 24 individuals (mean age: 45.3 years) recovering from COVID-19 was tested for cytokines, antibody titers and neuronal-enriched extra-cellular (nEV) protein cargo vehicles, at a median of 60 days (range: 30–103 days) from onset of symptoms [11]. They found that the cargo of the nEVs from all recovering COVID-19 patients, regardless of time after infection, was altered. However, it was unknown if these neurodegenerative proteins are transient or long-term; if this is the case, it may indicate continuing neuroinflammation or a signal to neurodegeneration [11]. In particular, a comparison of eight patients with neurological problems (mainly memory or cognition problems) to 16 patients with no neurological problems showed a positive association between post-COVID neurological manifestations and increased SARS-CoV-2 IgG antibody titers, increased IL-6 levels and co-morbidities [11]. Plasma IL-4 levels continued to be elevated in all 24 COVID-19 patients. IL-4 is involved in brain function, such as memory. Its elevation signals an ongoing neuroinflammation after COVID-19 infection that may influence neurological sequelae by altering nEV proteins. It is possible that patients recovering from COVID-19 may have occult neurological injury, while those with perceived neurologic symptoms may have more severe infection, as also indicated by the elevated SARS-CoV-2 antibody titers [11]. They also studied 12 pre-COVID-19 historical controls (mean age: 52.3 years) [11]. Protein markers of neuronal dysfunction including amyloid-beta, neurofilament light chain, neurogranin, total tau, and pT181-tau were all significantly increased in the nEVs of all participants recovering from COVID compared to pro-COVID-19 historical controls. A limitation of this study was the fact that neurological complaints were self-reported, with no neuropsychological testing conducted [11]. Larger studies are needed to assess if these findings are persistent and to find if patients without neurological complains are truly not affected long-term.

It is known that coronaviruses are neurotropic and may invade the blood-brain barrier and access the CNS through periphery or olfactory neurons. The hippocampus appears to be particularly vulnerable to infection, which may also contribute to post-infection memory deficit [58]. Wostyn proposed the hypothesis that post-COVID fatigue syndrome may result from damage to olfactory sensory neurons, causing a reduced outflow of cerebrospinal fluid (CSF) through the cribriform plate, and leading to congestion of the lymphatic system with subsequent toxic build-up within the CNS [59]. In addition, direct SARS-CoV-2 neuroinvasion has been proposed as a mechanism that may lead to persistent neurophychiatric complications [22], but it appears less likely on the basis of time that elapsed from initial infection.

Beyond inflammation, post-COVID fatigue may be attributed to lung dysfunction. A prospective observational three-month follow-up study of 76 patients (mean age: 41.3 years) found that serum troponin-I levels during the acute illness were significantly associated with the onset of fatigue after discharge (*p*-value = 0.008), while lymphopenia was significantly associated with chest tightness and palpitations on exertion after discharge (*p*-value = 0.004) [31]. In this series, the mean values of forced expiratory volume in 1 s (FEV1), forced vital capacity (FVC), FeV1/FVC, total lung capacity and diffusion capacity were all normal, however 42% of patients had mild pulmonary function abnormalities at three months after discharge [31]. A limitation of this study is that most patients (91%) had mild COVID-19 and no past medical history of lung function was available [31].

In a series of female patients, aged 26–50 years with symptoms compatible with post-COVID syndrome (of whom one had laboratory-confirmed COVID-19), all had orthostatic intolerance [60]. Cytokine storm is accompanied by the activation of the sympathetic system and a surge of catecholamines, which in turn trigger the production of IL-6 and other cytokines, and therefore increases inflammation injury [61]. An inflammatory-mediated impairment of the autonomous nervous system, resulting in orthostatic intolerance, has been proposed [60]. In a report of two cases of post-COVID depression, the authors showed an association between depression and interleukins, such as IL-6, which was independent of other causes of depression that occurred during the COVID-19 pandemic (e.g., due to isolation) [9]. Based on this finding, the authors suggested that the administration of specific medication to reduce the cytokine activity is justified, since the normalization of pro-inflammatory cytokines decreased depression, regardless of the use of anti-depressant treatment [9]. In a 69-year-old patient with focal refractory status epilepticus (RSE) six weeks after initial infection and recovery from severe COVID-19, the only notable laboratory findings at the time of the onset of focal RSE were the elevated serum inflammatory markers and CSF protein, IgG, and IgG index, reflecting breakdown of the blood-brain barrier and inflammation in the CNS [62]. The patient’s RSE was attributed to post-infectious inflammatory response, as indicated by the raised inflammatory markers [62]. Similarly, profound inflammation, as indicated by increased levels of the cytokines tumor necrosis factor (TNF)-α, IL-1, and IL-6, leading to cochlea cell stress, has been implicated in the sudden irreversible sensorineural hearing loss that occurred in a patient with severe complicated COVID-19 [11]. Direct virus invasion in the cochlea may also account for the hearing loss [11]. Vascular impairment, in association with vascular endothelial growth factor, IL-6 and TNFα, is also prominent in the inflammatory phase of acute respiratory distress syndrome (ARDS) and may account for post-COVID-19 pulmonary fibrosis, which is characterized by uncontrolled fibroproliferation in the context of dysregulated release of matrix metalloproteinases, leading to injury of endothelium and epithelium [63]. Likewise, infected monocytes and macrophages, which are part of the first cellular immune response to acute SARS-CoV-2 infection, may contribute to cytokine storm and massively migrate from lungs to tissues, and appear to contribute to post-COVID complications, including fibrosis, while their manipulation may open novel therapeutic perspectives [53,55,64]. High-resolution chest computed tomography has shown architectural distortion, interlobal septal thickening and traction bronchiectasis, compatible with fibrotic lung disease, in patients who continue to have hypoxia, even after three weeks of treatment, despite the improvement of their symptoms [63]. Likewise, a French prospective follow-up study of 478 hospitalized COVID-19 patients (mean age: 61 years), evaluated four months after discharge, found that 244 (51%) had at least one symptom that did not exist before COVID-19, mainly fatigue (31%), cognitive symptoms (21%), and new-onset dyspnea [4]. Lung computed tomography in 177 patients, including 97 former ICU patients, revealed abnormalities in 108 (63%) patients (mainly subtle ground-glass opacities) and fibrotic lesions in 33 (19%) patients, mainly among patients with ARDS [8]. In echocardiography, the left ventricular ejection fraction was < 50% in 8 (10%) of 83 ICU patients [8]. The authors recognize the absence of pre-COVID assessment in their cohort [8]. An Italian study of 238 patients (median age: 61 years; 59.7% men; mean of two co-morbidities) hospitalized for severe COVID-19, found that 128 (53.8%) of them had prolonged pulmonary impairment at four months post-discharge, as indicated by pulmonary function tests and diffusion lung capacity for carbon monoxide (D_LCO_), which in turn may account for several post-COVID symptoms [37].

Post-viral infection destruction of β-pancreatic cells can occur and trigger the onset of diabetes mellitus. It has been shown that SARS-CoV-2 can infect and replicate in human pancreatic islets, in association with reduced insulin-secreting granules in pancreatic β-cells and impaired glucose-stimulated insulin secretion [65], which may explain the deterioration of glycemic control observed in diabetic patients with COVID-19 necessitating exceptionally high doses of insulin [66], but also increase the risk for onset of diabetes after COVID-19 [67]. Potential pathways of injury of pancreatic β-cells include a profound proinflammatory cytokine response, leading to a chronic low-grade inflammationactivation of the renin-angiotensin-aldosterone system, through the SARS-CoV-2 target ACE2 receptor, which is abundant in pancreatic β-cells, and enhances autoimmunity in genetically predisposed individuals [67].

Lastly, a new multisystem inflammatory syndrome in children (MIS-C), in association with SARS-CoV-2 infection, has been reported [68]. The main findings in this rare, but severe, clinical syndrome include shock, cardiac dysfunction, gastrointestinal symptoms, dermatologic/mucocutaneous symptoms, and elevated inflammatory markers (CRP, IL-6, and fibrinogen levels) [68]. There is also evidence that adult patients of all ages may develop a MIS-C-like syndrome associated with SARS-CoV-2 infection [68]. In particular, several cases have been described in adults and the term multisystem inflammatory syndrome in adults (MIS-A) has been proposed [69]. The MIS-A syndrome is characterized by a wide spectrum of cardiovascular, gastrointestinal, dermatologic, and neurologic symptoms and a temporal association with SARS-CoV-2 infection, diagnosed either through RT-PCR or serologically, indicating recent infection [69]. In contrast to severe COVID-19, a distinct characteristic of this hyperinflammatory syndrome is the absence of severe respiratory illness [69]. Similar to MIS-C in children [68], a post-infectious inflammatory pathogenetic mechanism is indicated by the fact that in one third of cases, the diagnosis of SARS-CoV-2 infection is established through serology in the absence of a positive PCR test [69]. Persistent extra-pulmonary infection is also possible, since the virus has been detected in multiple organs, including the heart, liver, brain, kidneys, and gastrointestinal tract [69]. Additional proposed mechanisms for extrapulmonary dysfunction in COVID-19 include endothelial damage and thromboinflammation, dysregulated immune responses, and dysregulation of the renin-angiotensin-aldosterone system [69]. The interval between infection and development of MIS-A is unclear, adding to the uncertainty regarding whether MIS-A represents a manifestation of acute infection or an entirely post-acute phenomenon. In patients who reported typical COVID-19 symptoms before MIS-A onset, MIS-A was experienced approximately 2–5 weeks later [69]. However, eight MIS-A patients reported no preceding respiratory symptoms, making it difficult to estimate when initial infection occurred [69]. Given the high proportion of MIS-A patients with negative PCR testing, clinical guidelines recommend the use of both antibody and viral testing to assist with diagnosis [69]. In patients with atypical or late manifestations of SARS-CoV-2 infection, including MIS-A, positive antibody results might be crucial to augment clinical recognition of this condition and guide treatment [69]. In addition, the use of a panel of laboratory tests for inflammation, hypercoagulability, and organ damage (e.g., CRP, ferritin, D-dimer, cardiac enzymes, liver enzymes, and creatinine) might assist in the early identification and management of this COVID-19–associated condition [69]. Further research is needed to understand the pathogenesis and long-term effects of this newly described condition.

Endothelial cell infection through viremia is also a major pathogenic mechanism in acute COVID-19, contributing to thrombosis and bleeding (e.g., pulmonary emboli) [55]. Vascular dysfunction also appears to be implicated in post-COVID syndrome. Four weeks after PCR diagnosis of COVID-19, a 64-year-old woman developed cerebral hypoperfusion syndrome, along with both central (dizziness, brain fog) and peripheral (distal burning sensation) nervous system dysfunction, which are indicative of reduced orthostatic cerebral blood flow [70]. In this case, abnormal arterial vasoconstriction and immune-mediated dysfunction most probably caused cerebral autoregulatory failure; IVIG administration resulted in the subsidence of symptoms, which is consistent with an autoimmune mechanism, triggered by COVID-19 [70]. In addition, in a previously healthy 56-year-old man with persistent neurological disorders, including short-duration epileptic seizures, and deep depression almost six months after COVID-19, CNS MRI revealed numerous hyperintense focal areas in the periventricular and subcortical white matter and in semioval centers, compatible with gliotic outcomes in association with microvascular injuries [71]. Endothelial injury, either due to virus invasion or in the context of profound inflammation increased levels of coagulation factors, hypoxia, due to respiratory impairment or anti-platelel factor 4 (PF4)-immune complexes, has been implicated in acute COVID-19 pathogenesis, predisposing to coagulopathy and thromboembolic complications in large blood vessels and microcirculation [53,72,73]. Thromboembolism may also complicate the post-COVID period in the context of a hypercoagulate state [74]. An increased risk of developing pulmonary thromboembolism, deep vein thrombosis, and thrombosis in other systems manifested by active bleed, has been recorded in patients who have recovered from COVID-19 [74]. Follow-up of at least 30 days post-discharge is required, while patients at high-risk for thrombosis should receive anticoagulation medications for a prolonged period [74]. A recent study from Ireland found increased D-dimer levels (>500 ng/mL) in 25.3% of 150 COVID-19 patients, including 60 with a history of hospitalization, up to four months after initial diagnosis [75]. In this latter study, increased convalescent D-dimers were more common in COVID-19 patients who had required hospitalization, and in patients older than 50 years [75].

## 7. Future Challenges—Implications for Research

In view of the dynamic of the COVID-19 pandemic globally, it is expected that a large number of patients will seek healthcare in the coming months or years because of post-COVID morbidity [76,77]. Consensus-based guidelines for the diagnosis, classification, and management of this new clinical entity are urgently needed. In addition, registries to systemically follow-up COVID-19 patients should be issued to estimate the incidence, full clinical spectrum, and prognosis of patients with post-COVID syndrome in the long-term [5]. Although to our knowledge, there is no published study on the potential benefit of COVID-vaccination on post-COVID syndrome, while there are reports on the post-vaccination resolution of symptoms such as fatigue, shortness of breath, insomnia, muscle pain, and gastrointestinal symptoms [78,79]. Proposed hypotheses on how vaccines might improve post-COVID symptoms include: T cells, boosted by the vaccine, could eliminate a viral reservoir; the vaccine could trigger an increased immune response; or it could divert an inappropriate autoimmune response [77]. If this is the case, vaccination will be part of the solution with major clinical and public health implications. Meanwhile, it is essential to elucidate the multi-factorial pathogenesis of post-COVID syndrome and guide research toward the identification of markers for therapeutic and prognostic purposes.

## 8. Limitations

Information regarding post-COVID concerns mostly hospitalized COVID-19 cases. Another limitation is the absence of published evidence from nationwide COVID-19 registries with long-term follow-up. In addition, information provided in this article regarding the clinical spectrum of post-COVID syndrome is based on current evidence but may be expanded as more information becomes available in the future. Recall bias, or bias of over reporting severe or noisy cases, should be considered.

## 9. Conclusions

This is an overview of published information on post-COVID syndrome, focusing on its pathogenesis. The evolving data indicate a multi-factorial pathogenesis, namely inflammation, nervous system dysfunction, endothelial damage, and thromboembolism as the main pathogenetic mechanisms. It is expected that as the long-term complications of COVID-19 unfold, more evidence will be available to guide therapeutic management. Further research is needed in order to elucidate the incidence, clinical spectrum, pathogenesis, and prognosis of this new clinical entity. In the interim, standardization of definitions and a consensus on classification criteria are needed. Given the potential bias of published information, prospective registries for active follow-up of COVID-19 cases are essential to provide accurate estimations of the incidence, clinical spectrum, and prognosis of post-COVID syndrome.

## Figures and Tables

**Table 1:** Classifications of post-COVID syndrome *.

Becker et al. [18] (COVID-19 Clinic of the University of Cincinnati Medical Center)							
	Type 1	Type 2	Type 3		Type 4		Type 5
Initial symptoms	Variable ^a^	Mild	A	B	A	B	None
			Mild	Mild	None	None	
Duration of symptoms	Variable ^a^	>6 weeks	3–6 months	>6 months	Variable	Variable	N/A
Period of quiescence	No	No	Yes	Yes	No	No	N/A
Delayed onset of symptoms	No	No	No		Yes ≥3 months	Yes ≥6 months	Yes
**Amenta et al. [22]** **(Department of Medicine, Baylor College of Medicine, Houston)**							
MIS: >3 weeks from suspected infection							
Persisting symptoms: >3 weeks from symptom onset							
Organ dysfunction: Time of hospital discharge (if >3 weeks after symptom onset)							
**Fernández-de-las-Peñas et al. [23]** **(Department of Physical Therapy, Occupational Therapy, Physical Medicine and Rehabilitation, Universidad Rey Juan Carlos)**							
Transition Phase:Symptoms potentially associated with acute COVID-19: symptoms up to 4–5 weeks							
Phase 1:Acute post-COVID symptoms: symptoms from week 5 to week 12							
Phase 2:Long post-COVID symptoms: symptoms from week 12 to week 24							
Phase 3:Persistent post-COVID symptoms: symptoms lasting more than 24 weeks							

^a^ Correlate with the severity of the initial infection, number of organ system injured and pre-existing medical conditions * as of 30 March 2020.

## References

[1] World Health Organization Coronavirus Disease (COVID-19) Situation Reports. https://www.who.int/emergencies/situation-reports.

[2] Lagier J.C., Million M., Gautret P., Colson P., Cortaredona S., Giraud-Gatineau A. (2020). Outcomes of 3737 COVID-19 patients treated with hydroxychloroquine/azithromycin and other regimens in Marseille, France: A retrospective analysis. Travel Med. Infect. Dis..

[3] Eythorsson E., Helgason D., Ingvarsson R.F., Bjornsson H.K., Olafsdottir L.B., Bjarnadottir V. (2020). Clinical spectrum of coronavirus disease 2019 in Iceland: Population based cohort study. BMJ.

[4] Maltezou H.C., Raftopoulos V., Vorou R., Papadima K., Mellou K., Spanakis N., Kossyvakis A., Gioula G., Exindari M., Froukala E. (2021). Association between upper respiratory tract viral load, comorbidities, disease severity, and outcome of patients with SARS-CoV-2 infection. J. Infect. Dis..

[5] Pavli A., Theodoridou M., Maltezou H.C. (2021). Post-COVID syndrome: Incidence, clinical spectrum, and challenges for primary healthcare professionals. Arch. Med. Res..

[6] Havervall S., Rosell A., Phillipson M., Mangsbo S.M., Nilsson P., Hober S., Thelin C. (2021). Symptoms and functional impairment assessed 8 months after mild COVID-19 among health care workers. JAMA.

[7] Patient-Led Research Collaborative Report: What Does COVID-19 Recovery Actually Look Like? An Analysis of the Prolonged COVID-19 Symptoms Survey by Patient-Led Research Team. https://patientresearchcovid19.com/research/report-1/.

[8] Morin L., Savale L., Pham T., Colle R., Figueiredo S., Harrois A., Gasnier M., Lecoq A.L., Meyrignac O., Writing Committee for the COMEBAC Study Group (2021). Four-month clinical status of cohort of patients after hospitalization for COVID-19. JAMA.

[9] Alpert O., Begun L., Garren P., Solhkhah R. (2020). Cytokine storm induced new onset depression in patients with COVID-19. A new look into the association between depression and cytokines—Two case reports. Brain Behav. Immun. Health.

[10] Dicpinigaitis P.V., Canning B.J. (2020). Is there (will there be) a post-COVID-19 chronic cough?. Lung.

[11] Koumpa F.S., Forde C.T., Manjaly J.G. (2020). Sudden irreversible hearing loss post COVID-19. BMJ Case Rep..

[12] Sun B., Tang N., Peluso M.J., Iyer N.S., Torres L., Donatelli J.L., Munter S.E., Nixon C.C., Rutishauser R.L., Rodriguez-Barraquer I. (2021). Characterization and biomarker analyses ofpost-COVID-19 complications and neurological manifestations. Cells.

[13] Wiersinga W.J., Rhodes A., Cheng A.C., Peacock S.J., Prescott H.C. (2020). Pathophysiology, transmission, diagnosis, and treatment of coronavirus disease 2019 (COVID-19): A review. JAMA.

[14] Greenhalgh T., Knight M., A’Court M., Buxton M., Husain L. (2020). Management of post-acute COVID-19 in primary care. BMJ.

[15] Tenforde M., Kim S., Lindsell C., Billig Rose E., Shapiro N.I., Files D.C., Gibbs K.W., Erickson H.L., Steingrub J.S., Smithline H.A. (2020). Symptom duration and risk factors for delayed return to usual health among outpatients with COVID-19 in a multistate health care systems network-United States, March–June 2020. MMWR Morb. Mortal. Wkly. Rep..

[16] Covid-19-Long-Term-Health-Effects. https://www.gov.uk/government/publications/covid-19-long-term-health-effects/covid-19-long-term-health-effects.

[17] Carvalho-Schneider C., Laurent E., Lemaignen A., Lemaignen A., Beaufils E., Bourbao-Tournois C., Laribi S., Flament T., Ferreira-Maldent N., Bruyère F. (2021). Follow-up of adults with noncritical COVID-19 two months after symptom onset. Clin. Microbiol. Infect..

[18] Becker R.C. (2021). COVID-19 and its sequelae: A platform for optimal patient care, discovery and training. J. Thromb. Thrombolysis.

[19] Nalbandian A., Sehgal K., Gupta A., Madhavan M.V., McGroder C., Stevens J.S., Cook J.R., Nordvig A.S., Shalev D., Sehrawat T. (2021). Post-acute COVID syndrome. Nat. Med..

[20] Al-Jahdhami I., Al-Naamani K., Al-Mawali A. (2021). The post-acute COVID-19 syndrome (long COVID). Oman Med. J..

[21] Scordo K.A., Richmond M.M., Munro N. (2021). Post-COVID-19 syndrome: Theoretical basis, identification, and management. AACN Adv. Crit. Care.

[22] Amenta E.M., Spallone A., Rodriguez-Barradas M.C., El Sahly H.M., Atmar R.L., Kulkarni P.A. (2020). Post-acute COVID-19: An overview and approach to classification. Open Forum Infect. Dis..

[23] Fernandez-de-Las-Penas C., Palacios-Cena D., Gomez-Mayordomo V., Cuadrado M.L., Florencio L.L. (2021). Defining post-COVID symptoms (post-acute COVID, long COVID, persistent post-COVID): An integrative classification. Int. J. Environ. Res. Public Health.

[24] Huang C., Huang L., Wang Y., Li X., Ren L., Gu X., Kang L., Guo L., Liu M., Zhou X. (2021). 6-month consequences of COVID-19 in patients discharged from hospital: A cohort study. Lancet.

[25] Simani L., Ramezani M., Darazam I.A., Sagharichi M., Aalipour M.A., Ghorbani F., Pakdaman H. (2021). Prevalence and correlates of chronic fatigue syndrome and post-traumatic stress disorder after the outbreak of the COVID-19. J. Neurovirol..

[26] Carfi A., Bernabei R., Landi R. (2020). Persistent symptoms in patients after acute COVID-19. JAMA.

[27] Addison A.B., Wong B., Ahmed T., Macchi A., Konstantinidis I., Huart C., Frasnelli J., Fjaeldstad A.W., Ramakrishnan V.R., Rombaux P. (2021). Clinical olfactory working group consensus statement on the treatment ofpostinfectious olfactory dysfunction. J. Allergy Clin. Immunol..

[28] Le Bon S.D., Pisarski N., Verbeke J., Prunier L., Cavelier G., Thill M.-P., Rodriguez A., Dequanter D., Lechien J.R., Le Bon O. (2020). Psychophysical evaluation of chemosensory functions 5 weeks after olfactory loss due to COVID-19: A prospective cohort study on 72 patients. Eur. Arch. Oto-Rhino-Laryngol..

[29] Moreno-Pérez O., Merino E., Leon-Ramirez J.M., Prunier L., Cavelier G., Thill M.P., Rodriguez A., Dequanter D., Lechien J.R., Le Bon O. (2021). COVID19-ALC research Post-acute COVID-19 Syndrome. Incidence and risk factors: A Mediterranean cohort study. J. Infect..

[30] Garg P., Arora U., Kumar A., Wig N. (2021). The “post-COVID” syndrome: How deep is the damage?. J. Med. Virol..

[31] Liang L., Yang B., Jiang N., Fu W., He X., Zhou Y., Ma W.L., Wang X. (2020). Three-month follow-up study of survivorsof Coronavirus Disease 2019 after discharge. J. Korean Med. Sci..

[32] Moldofsky H., Patcai J. (2011). Chronic widespread musculoskeletal pain, fatigue, depression and disordered sleep in chronic post-SARS syndrome; a case-controlled study. BMC Neurol..

[33] Willi S., Luthold R., Hunt A., Hanggi N.V., Sejdiu D., Scaff C., Bender N., Staub K., Schlagenhauf P. (2021). COVID-19 sequelae in adults aged less than 50 years: A systemic review. Travel. Med. Infect. Dis..

[34] Halpin S.J., McIvor C., Whyatt E.G., Adams A., Harvey O., McLean L., Walshaw C., Kemp S., Corrado J., Singh R. (2021). Post-discharge symptoms and rehabilitation needs in survivors of COVID-19 infection: A cross-sectional evaluation. J. Med. Virol..

[35] Garrigues E., Janvier P., Kherabi Y., Le Bot A., Hamon A., Gouze H., Doucet L., Berkani S., Oliosi E., Mallart E. (2020). Post-discharge persistent symptoms and health-related quality of life after hospitalization for COVID-19. J. Infect..

[36] Davis H.E., Assaf G.S., McCorkell L., Wei H., Low R.J., Re’em Y., Redfield S., Austin J.P., Akrami A. Characterizing Long COVID in an International Cohort: 7 Months of Symptoms and Their Impact. https://www.medrxiv.org/content/10.1101/2020.12.24.20248802v2.

[37] Bellan M., Soddu D., Balbo P.E., Baricich A., Zeppegno P., Avanzi G.C., Baldon G., Bartolomei G., Battaglia M., Battistini S. (2021). Respiratory and psychophysical sequelae among patients withCOVID-19four months after hospital discharge. JAMA Netw. Open.

[38] Chopra V., Flanders S.A., O’Malley M., Malani A.N., Prescott H.C. (2021). Sixty-day outcomes among patients hospitalized with COVID-19. Ann. Intern. Med..

[39] Saeed S., Tadic M., Larsen T.H., Grassi G., Mancia G. (2021). Coronavirus disease 2019 and cardiovascular complications; focused clinical review. J. Hypertens..

[40] Mitrani R.D., Dabas N., Goldberger J.J. (2020). COVID-19 cardiac injury: Implications for long-term surveillance and outcomes in survivors. Heart Rhythm..

[41] Alemanno F., Houdayer E., Parma A., Spina A., Del Forno A., Scatolini A., Angelone S., Brugliera L., Tettamanti A., Beretta L. (2021). COVID-19 cognitive deficits after respiratory assistance in the subacute phase: ACOVID-rehabilitation unit experience. PLoS ONE.

[42] Soloveva N.V., Makarova E.V., Kichuk I.V. (2021). Coronavirus syndrome: COVID-19 psychotrauma. Eur. J. Transl. Myol..

[43] Chang M.C., Park D. (2020). Incidence of post-traumatic stress disorder after Coronavirus Disease. Health Care.

[44] Raahimi M.M., Kane A., Moore C.E., Alareed A.W. (2021). Late onset of Guillain-Barre syndrome following SARS-CoV-2 infection: Part of long COVID-19 syndrome?. BMJ Case Rep..

[45] Camdessanche J.P., Morel J., Pozzetto B., Paul S., Tholance Y., Botelho-Nevers E. (2020). COVID-19 may induce Guillain-Barré syndrome. Rev. Neurol..

[46] Emamikhah M., Babadi M., Mehrabani M., Jalili M., Pouranian M., Daraie P., Mohaghegh F., Aghavali S., Zaribafian M., Rohani M. (2021). Opsoclonus-myoclonus syndrome, a post-infectious neurologic complication of COVID-19: Case series and review of literature. J. Neurovirol..

[47] Scoppettuolo P., Borrelli S., Naeije G. (2020). Neurological involvement in SARS-CoV-2 infection: A clinical systematic review. Brain Behav. Immun. Health.

[48] Shahali H., Ghasemi A., Farahani R.H., Nezami Asl A., Hazrati E. (2021). Acute transverse myelitis after SARS-CoV-2 infection: A rare complicated case of rapid onset paraplegia. J. Neurovirol..

[49] Kilbertus S. (2021). Acute transverse myelitis attributed to SARS-CoV-2 infection presenting as impaired mobility: A case report. CJEM.

[50] Xia X., Wang Y., Zheng J. (2021). COVID-19 and Alzheimer’s disease: How one crisis worsens the other. Transl. Neurodegener..

[51] Leta V., Rodríguez-Violante M., Abundes A., Rukavina K., Teo J.T., Falup-Pecurariu C., Irincu L., Rota S., Bhidayasiri R., Storch A. (2021). Parkinson’s disease and post-COVID-19 syndrome: The Parkinson’s long-COVID spectrum. Mov. Disord..

[52] Dennis A., Wamil M., Alberts J., Oben J., Cuthbertson D.J., Wootton D., Crooks M., Gabbay M., Brady M., Hishmeh L. (2021). Multiorgan impairment in low-risk individuals with post-COVID-19 syndrome: A prospective, community-based study. BMJ Open.

[53] Gautret P., Million M., Jarrot P.A., Camoin-Jau L., Colson P., Fenollar F., Leone M., La Scola B., Devaux C., Gaubert J.Y. (2020). Natural history of COVID-19 and the rapeutic options. Expert Rev. Clin. Immunol..

[54] Trougakos I.P., Stamatelopoulos K., Terpos E., Tsitsilonis O.E., Aivalioti E., Paraskevis D., Kastritis E., Pavlakis G.N., Dimopoulos M.A. (2021). Insights to SARS-CoV-2 life cycle, pathophysiology, and rationalized treatments that target COVID-19 clinical complications. J. Biomed Sci..

[55] Stratton C.W., Tang Y.W., Lu H. (2021). Pathogenesis-directed therapy of 2019 novel coronavirus disease. J. Med.Virol..

[56] Ortelli P., Ferrazzoli D., Sebastianelli L., Engl M., Romanello R., Nardone R., Bonini I., Koch G., Saltuari L., Quartarone A. (2021). Neuropsychological and neurophysiological correlates of fatigue in post-acute patients with neurological manifestations of COVID-19: Insights into a challenging symptom. J. Neurol. Sci..

[57] Garcia-Oscos F., Salgado H., Hall S., Thomas F., Farmer G.E., Jorge Bermeo J., Galindo L.C., Ramirez R.D., D’Mello S., Rose-John S. (2012). The stress-induced cytokine interleukin-6 decreases the inhibition/excitation ratio in the rat temporal cortex via trans-signaling. Biol. Psychiatry.

[58] Ritchie K., Chan D., Watermeyer T. (2020). The cognitive consequences of the COVID-19 epidemic: Collateral damage?. Brain Commun..

[59] Wostyn P. (2021). COVID-19 and chronic fatigue syndrome: Is the worst yet to come?. Med. Hypotheses.

[60] Dani M., Dirksen A., Taraborrelli P., Torocastro M., Panagopoulos D., Sutton R., Lim P.B. (2021). Autonomic dysfunction in ’long COVID’: Rationale, physiology and management strategies. Clin. Med..

[61] Konig M.F., Powell M., Staedtke V., Bai R.Y., Thomas D.L., Fischer N., Hug S., Khalafallah A.M., Koenecke A., Xiong R. (2020). Preventing cytokine storm syndrome in COVID-19 using α-1 adenergic receptor antagonists. J.Clin. Investig..

[62] Carroll E., Neumann H., Aguero-Rosenfeld M.E., Lighter J., Czeisler B.M., Melmed K., Lewis A. (2020). Post-COVID-19 inflammatory syndrome manifesting as refractory status epilepticus. Epilepsia.

[63] Tale S., Ghosh S., Meitei S.P., Kolli M., Garbhapu A.K., Pudi S. (2020). Post-COVID-19 pneumonia pulmonary fibrosis. QJM.

[64] Boumaza A., Gay L., Mezouar S., Bestion E., Diallo A.B., Michel M., Desnues B., Raoult D., La Scola B., Halfon P. (2021). Monocytes and macrophages, targets of SARS-CoV-2: The clue for Covid-19 immunoparalysis. J. Infect. Dis..

[65] Muller J.A., Grob R., Conzelmann C., Kruger J., Merle U., Steinhart J., Weil T., Koepke L., PrelliBozzo C., Read C. (2021). SARS-CoV-2 infects and replicates in cells of the human endocrine and exocrine pancreas. Nat. Metab..

[66] Hayden M.R. (2020). An immediate and long-term complication of COVID-19 may be type 2 diabetes mellitus: The central role of β-cell dysfunction, apoptosis and exploration of possible mechanisms. Cells.

[67] Sathish T., Tapp R.J., Cooper M.E., Zimmer P. (2021). Potential metabolic and inflammatory pathways between COVID-19 and new-onset diabetes. Diabetes Metab..

[68] Abrams J.Y., Godfred-Cato S.E., Oster M.E., Chow E.J., Koumans E.H., Bryant B., Leung J.W., Belay E.D. (2020). Multisystem inflammatory syndrome in children associated with severe acute respiratory syndrome coronavirus 2: A systemic review. J. Pediatr..

[69] Bamrah Morris S., Schwartz N., Patel P., Abbo L., Beauchamps L., Balan S., Lee E.H., Paneth-Pollak R., Geevarughese A., Lash M.K. (2020). Case series of multisystem inflammatory syndrome in adults associated with SARS-CoV-2 infection-United Kingdom and United States, March–August 2020. Morbid/Mortal. Wkly. Rep..

[70] Novak P. (2020). Post COVID-19 syndrome associated with orthostatic cerebral hypoperfusion syndrome, small fiber neuropathy and benefit of immunotherapy: A case report. Eneurological Sci..

[71] Nuzzo D., Cambula G., Bacile I., Rizzo M., Galia M., Mangiapane P., Picone P., Giacomazza D., Scalisi L. (2021). Long-term brain disorders in post Covid-19 neurological syndrome (PCNS) patient. Brain Sci..

[72] Nazy I., Jevtic S.D., Moore J.C., Huynh A., Smith J.W., Kelton J.G., Arnold D.M. (2021). Platelet-activating immune complexes identified in critically ill COVID-19 patients suspected of heparin-induced thrombocytopenia. J. Thromb. Haemost..

[73] Brodard J., Kremer Hovingam J.A., Fontanam P., Studt J.D., Gruel Y., Greinacher A. (2021). COVID-19 patients often show high-titer non-platelet-activating anti-PF4/heparin IgG antibodies. J. Thromb. Haemost..

[74] Kumar M.A., Krishnaswamy M., Arul J.N. (2021). Post COVID-19 sequelae: Venous thromboembolism complicated by lower GI bleed. BMJ Case Rep..

[75] Townsend L., Fogarty H., Dyer A., Martin-Loeches I., Bannan C., Nadarajan P., Bergin C., O’Farrelly C., Conlon N., Bourke N.M. (2021). Prolonged elevation of D-dimer levels in convalescent COVID-19 patients is independent of the acute phase response. J. Thromb. Haemost..

[76] Ladds E., Rushforth A., Wieringa S., Taylor S., Rayner C., Husain L., Greenhalgh T. (2020). Persistent symptoms after COVID-19: Qualitative study of 114 “long COVID” patients and draft quality principles for services. BMC Health Serv. Res..

[77] Davido B., Seang S., Tubiana R., de Truchis P. (2020). Post-COVID-19 chronic symptoms: A postinfectious entity?. Clin. Microbiol. Infect..

[78] Washington Post Some Long-Haul COVID-19 Patients Say Their Symptoms are Subsiding after Getting Vaccines. https://www.washingtonpost.com/health/long-haul-covid-vaccine/2021/03/16/6effcb28-859e-11eb-82bc-e58213caa38e_story.html.

[79] Medscape Pediatrics Some with Long COVID See Relief after Vaccination. https://www.medscape.com/viewarticle/947592.

